# Childhood maltreatment, depression and their link to adult economic burdens

**DOI:** 10.3389/fpsyt.2022.908422

**Published:** 2022-08-22

**Authors:** Julia Petersen, Ann-Christin Schulz, Elmar Brähler, Cedric Sachser, Jörg M. Fegert, Manfred E. Beutel

**Affiliations:** ^1^Department of Psychosomatic Medicine and Psychotherapy, University Medical Center, Johannes Gutenberg University Mainz, Mainz, Germany; ^2^Department of Psychiatry and Psychotherapy, Leipzig University Medical Center, Leipzig, Germany; ^3^Department of Child and Adolescent Psychiatry and Psychotherapy, Ulm University, Ulm, Germany

**Keywords:** adverse childhood experiences (ACE), depression, economic burdens, poverty, unemployment, education

## Abstract

**Background:**

Adult depression is a common consequence of adverse childhood experiences. There is also a higher likelihood of being affected by economic burdens after having experienced a traumatic event in childhood. As depression has been associated with economic burden, these long-term sequelae of childhood adversity are likely to interact.

**Goals:**

We investigated depression and economic consequences, such as unemployment, lower level of education, lower income as long-term sequelae of adverse childhood experiences in adulthood and their interaction.

**Methods:**

Childhood Maltreatment was measured by the German version of the Adverse Childhood Experience (ACE) questionnaire. Depression was measured by the Patient Health Questionnaire (PHQ-2). Logistic regressions were applied to investigate the risks of suffering economic burdens, with depression as a moderator.

**Results:**

Depressive symptoms increased with the number of ACEs and were highest in those reporting four or more ACEs, especially amongst those who experienced sexual and emotional abuse, as well as neglect. Moderation analysis showed a significant effect of depression increasing almost all economic burdens. Migration background additionally increased the risk of unemployment and working in a blue-collar job. Female gender decreased the risk of unemployment and working in a blue-collar job, but increased the risk of low income and part-time employment.

**Conclusion:**

The moderation effect of depression increased the negative impact of exposure to multiple ACEs on economic life in adulthood. Prevention of ACEs and early intervention are needed to prevent the mental health and economic consequences.

## Introduction

Adverse Childhood Experiences (ACEs) are a common phenomenon. In representative studies up to 43.7% of respondents of the German population reported having experienced at least one adverse childhood experience, which includes emotional, physical and sexual abuse, as well as neglect and household dysfunction, such as domestic violence and drug abuse (1). Most respondents who have experienced an adverse event in their childhood have also been exposed to at least another adverse event, with 8.9% reporting at least four or more ACEs (1). Studies repeatedly showed that having experienced one ACE increased the risk of experiencing another one. For example, Clemens et al. (2) reported that having witnessed domestic violence increased the risks of sexual abuse by 4.4 times, of emotional neglect 6.5 times, of physical abuse 8.8 times, and the risk of physical neglect even by 10.3 times. In another study, Clemens et al. (3) found that having grown up in a family with mental illness or substance abuse history increased the risk of child maltreatment 5.07–5.63 times and the risk of physical abuse by 6.81 times as well as the risk of physical neglect by 6.91 times.

Additionally, studies have also found strong associations between the occurrence of ACEs and negative psychological, emotional, social, health and economic outcomes (1, 4–8). Adverse events in childhood have been consistently identified as risk factors for depression in the past decades (3, 9–11). Being exposed to any ACE increased the likelihood of experiencing depression by almost three (12) to four (1) times. A cumulative experience of multiple ACEs lead to stronger associations, increasing the ratios for depression up to OR = 7.8 (95% confidence interval: [5.45; 11.13]) (1). Furthermore, having experienced traumatic experiences in childhood has been associated with higher risks of anxiety, negative repetitive thinking, dysfunctional metacognitive beliefs, stress systems dysregulations, as well as advanced biological aging (13–16).

According to stress sensitization theory, having experienced adversity in childhood can reduce a person's coping ability in stressful times, leading to greater reactivity and more depressive reactions compared to people who did not experience such events (17). This may lead to a so called “ripple effect” from early consequences of childhood trauma, such as, for example, an increased risk of lower educational achievement due to increased stress and/or mental health issues, to a decrease in one's economic productivity in later adult life. This includes increased unemployment, reduced working hours, working lower paid jobs, or permanent sick leave and may contribute ot negative economic long-term consequences and lower social class membership (3, 4, 6, 18–22). More studies described a “greater risk of sickness absence, having less assets, requiring income related support, experiencing financial insecurity and belonging to lower social class (odds ratios ranged from 1.73 to 2.98)” [(23), p. 129]. Fergusson et al. (24) also showed that lower gross income and welfare dependency were significantly associated with childhood sexual abuse, though the effect on income became insignificant when covariates were added. This effect remained significant among women, maybe because they are more likely to experience sexual abuse than men (22). Thus, gender seems to play a significant role in the manifestation of economic burdens for people with childhood trauma. For example, even after controlling for family SES and other variables, a significant negative effect of abuse on employment emerges, but only for women (20). Additionally, it has been observed that women in particular face a greater risk of suffering from economic stress later in life following a variety of ACEs (6). Physical maltreatment of women was significantly associated with reduced SES (25, 26). In contrast, in another study, men reported lower income and working hours following severe physical abuse in childhood; the effect was not significant for women (27).

In addition to these personal economic consequences following the experience of childhood maltreatment, researchers also began to evaluate the overall average economic lifetime costs of nonfatal childhood trauma and maltreatment in terms of medical costs, productivity losses and even in criminal justice costs. Fang et al. (28), for example, estimated total costs of all childhood trauma victims in the US as approximately $124 billion in 2008. Peterson et al. (29) updated these estimates from $210,012 to around $830,928 per non-fatal child maltreatment victim across their lifetimes, which raises the total costs of all non-fatal maltreatment cases to $428 billion. For Germany, the estimates of the same year ranged between 11.1 and 28.8 billion Euros (30).

As most of the current research is on non-German samples, this study aims to add to the growing research on the association between adverse childhood experiences and negative economic (as well as academic) outcomes in later life for Germany to understand better this complex interplay and to potentially aid in finding solutions to this big social, mental health and economic issue for a considerable part of the German population. We hypothesize, based on existing research, that ACEs negatively affect a person's mental health, increasing the risk of depression. We further hypothesize, that there is also an association between ACEs and economic burdens, such as lower levels of education and unemployment. Finally, due to the abovementioned “ripple effect” ACEs have on depression and depression on economic outcomes, we hypothesize that the associations between ACEs and economic burdens are moderated by depression. Finally, we assume gender effects regarding the negative psychological and economic outcomes.

## Materials and methods

### Data

A nationwide representative survey conducted by the independent institute for polling and social research (USUMA Berlin) was performed between December 2020 and February 2021. In total, 2,519 participants were included. However, we excluded all participants under the age of 25 since, by this time, they have finished their early adulthood stage and typically have become economically independent. This left us with a sample of 2,288 respondents (90.8% of the total sample).

### Measures

Sociodemographic information such as age, level of education, occupational status, times of unemployment, current occupation, net household income and migration background were asked of all participants of this study. Additionally, the equivalized income was calculated according to the Organization for Economic Co-operation and Development (OECD) (31). Adverse childhood experiences were surveyed using the German version of the ACE Questionnaire which consists of 10 items that inquire about child maltreatment and problems at home in childhood using yes/no answer categories (4, 32). Depression was measured using the Patient Health Questionnaire-4 (PHQ-4) which includes two items from the PHQ-9 and two items from the General Anxiety Disorder Screener (GAD-7) (33). Study participants were asked to assess how much they were impacted by the symptoms during the past 2 weeks on a 4-point scale ranging from 1 = *not at all* to 4 = *almost every day*. The subscale of PHQ-4 (PHQ-2) exhibits sufficient reliability (Cronbach's alpha = 0.72). In order to estimate the prevalence of depression, we used the validated cut-off value of ≥3 (34).

### Statistical analysis

First, we examine if we find a cumulative effect among the ACEs on depression by correlating the frequencies of ACEs (0, 1, 2, 3, 4+) with depression (PHQ-2), and with economic burdens [lower level of education, occupational status, blue collar occupation, frequency of unemployment and having an equivalized income of less than 1,126€, which is defined to be the 2020 at-risk-of-poverty threshold in Germany by the German Federal Office of Statistics (35)] using odds ratios. Second, we performed structural equation modeling (SEM) with the same variables as outcomes and added interaction terms to test if depression moderates the associations between ACEs and economic burdens. Before computing the interaction terms, we centered the predictors around their means (36). We then controlled for gender and migration background.

All calculations were performed using the statistical program R (version 4.1.2, packages: psych, naniar, poLCA, tidyLPA, tidyverse, lavaan).

## Results

### Demographics

One thousand ninety-one (47.7%) of the respondents were male with a mean age of 53.3 years (SD = 16.1). The full description of the sample can be found in Table 1. One thousand five hundred twenty-nine (66.8%) of the respondents reported having suffered no ACEs, 759 (33.2%) reported at least one. Compared to respondents without ACEs, more participants who reported ACEs had no academic degree (4.4 vs. 1.2%, *p* < 0.001). Furthermore, they had graduated less from secondary school (middle educational level; 39.3 vs. 45.4%, *p* = 0.007) and were less likely to hold a university degree (9.1 vs. 12.7%, *p* = 0.013). Regarding their current occupational status, respondents with ACEs were less likely employed full-time (37.7 vs. 49.0%, *p* < 0.001) but rather part-time employed (14.1 vs. 9.5%, *p* = 0.001) or currently unemployed (9.6 vs. 4.1%, *p* < 0.001) than their counterparts. Additionally, they were also more likely to have been unemployed at least twice (twice: 17.4 vs. 11.4%, *p* < 0.001; three times: 9.0 vs. 5.4%, *p* = 0.002; four times or more: 10.1 vs. 4.4%, *p* < 0.001). Participants with ACEs more frequently reported to be working blue collar jobs than participants without ACEs (26.8 vs. 22.7%, *p* = 0.039) and their mean household income was significantly lower (2,532.5 [SD = 1,538.3] vs. 2,859.5 [SD = 1,569.4], *p* < 0.001). Respondents with ACEs were more likely to have a migration background (15.5 9.7%, *p* < 0.001). And finally, they also exhibited significantly more likely symptoms of depression (15.9 3.0%, *p* < 0.001).

**Table 1:** Descriptive overview of social demographics of respondents who reported ACEs and those who did not.

	**Sample** ***N* = 2,288**	**With ACE** ***N* = 759, 33.2%**	**No ACE** ***N* = 1,529, 66.8%**	***p*-Value**
Gender (men)	1,091 (47.7%)	360 (47.4%)	731 (47.8%)	0.900
Age
25–35	364 (15.9%)	120 (15.8%)	244 (16.0%)	0.976
36–45	399 (17.4%)	134 (17.7%)	265 (17.3%)	0.894
46–55	411 (18.0%)	154 (20.3%)	257 (16.8%)	**0.047**
56–65	487 (21.3%)	150 (19.7%)	337 (22.1%)	0.230
66–75	390 (17.0%)	125 (16.5%)	265 (17.3%)	0.647
>75	237 (10.4%)	76 (10.0%)	161 (10.5%)	0.757
Education
Without a degree	52 (2.3%)	33 (4.4%)	19 (1.2%)	**0.000**
Secondary general school	651 (28.7%)	236 (31.4%)	415 (27.3%)	**0.047**
Secondary school	984 (43.3%)	295 (39.3%)	689 (45.4%)	**0.007**
Academic secondary school	318 (14.0%)	118 (15.7%)	200 (13.2%)	0.114
Tertiary education	261 (11.5%)	68 (9.1%)	193 (12.7%)	**0.013**
Other	4 (0.2%)	1 (0.1%)	3 (0.2%)	1.000
Occupational status
Full-time employment	1,028 (45.2%)	285 (37.7%)	744 (49.0%)	**0.000**
Part-time employment	252 (11.1%)	107 (14.1%)	145 (9.5%)	**0.001**
Unemployed	135 (5.9%)	73 (9.6%)	62 (4.1%)	**0.000**
Other	860 (37.8%)	292 (38.6%)	568 (37.4%)	0.616
Frequency of unemployment
0	1,168 (51.7%)	300 (40.2%)	868 (57.6%)	**0.000**
1	498 (22.1%)	174 (23.3%)	324 (21.2%)	0.337
2	302 (13.4%)	130 (17.4%)	172 (11.4%)	**0.000**
3	149 (6.6%)	67 (9.0%)	82 (5.4%)	**0.002**
≥4	140 (6.2%)	75 (10.1%)	65 (4.4%)	**0.000**
Occupation
Never been employed	28 (1.2%)	12 (1.6%)	16 (1.1%)	0.360
Blue collar workers	545 (24.1%)	200 (26.8%)	345 (22.7%)	**0.039**
White collar workers	1,350 (59.6%)	429 (57.4%)	921 (60.7%)	0.147
Civil servants	113 (5.0%)	33 (4.4%)	80 (5.3%)	0.437
Other	228 (10.1%)	73 (9.8%)	155 (10.2%)	0.797
Net household income (in Euro)	2,751.8 (1,566.4)	2,532.5 (1,538.3)	2,859.5 (1,569.4)	**0.000**
Equalized household income
≤ 1,000€	221 (9.9%)	108 (14.6%)	113 (7.5%)	**0.000**
≤ 2,000€	1,055 (47.1%)	363 (49.2%)	692 (46.1%)	0.179
≤ 3,000€	613 (27.3%)	177 (24.0%)	436 (29.0%)	**0.014**
>3,000€	351 (15.7%)	90 (12.2%)	261 (17.4%)	**0.002**
Migration background (yes)	267 (11.7%)	118 (15.5%)	149 (9.7%)	**0.000**
Depression (yes)	166 (7.3%)	120 (15.9%)	46 (3.0%)	**0.000**

*Significant (p ≤ 0.05) group differences in bold. p-Values were calculated using Pearson's Chi-squared test*.

*Depression according to PHQ-2 cut-off at ≥3*.

A total of 262 (11.5%) reported to have experienced one ACE, 138 (6.0%) two, 119 (5.2%) three, and 240 (10.5%) four or more ACEs. Emotional abuse was cited the most (363, 15.9%), followed by parental separation or divorce (324, 14.2%), physical abuse (288, 12.6%), substance abuse in the home (285, 12.5%), and emotional neglect (283, 12.4%). Incarceration of a family member (67, 2.9%) and sexual abuse (86, 3.8%) were mentioned least frequently.

### Associations between number of ACEs, depression and economic burdens

For individual ACEs, as well as multiple ACEs, we found significant moderate to strong associations with depression, income, level of education, current unemployment, blue collar education and higher risk of unemployment (see Table 2). The associations between the individual ACEs and depression ranged from OR = 2.80 (parental separation) to OR = 6.35 (sexual abuse). However, the highest associations were found for the cumulation of ACEs (ACE load) on depression. Additionally, no ACEs showed a significantly lower chance of depression (OR = 0.17) compared to the occurrence of four or more ACEs (OR = 10.20).

**Table 2:** Prevalence of combined adverse childhood experiences (ACEs), odds ratios and 95% confidence intervals for depression, income, education, working hours, occupation, and risk of unemployment.

	**Total sample**	**Depression^a^**	**Equivalized income ≤ 1,126€**	**Lower level of education^b^**	**Part-time working**	**Currently unemployed**	**Blue collar occupation**	**Higher risk of unemployment^c^**
**Number of ACEs (*****N*** **=** **2,288)**								
0	1,529 (66.8%)	1	1	1	1	1	1	1
1	262 (11.5%)	**3.57** [2.14–5.86]	**1.56** [1.06–2.26]	0.90 [0.66–1.21]	1.28 [0.83–1.91]	1.55 [0.85–2.66]	0.98 [0.71–1.33]	1.37 [0.90–2.02]
2	138 (6.0%)	**4.57** [2.67–8.55]	**1.71** [1.03–2.73]	0.80 [0.52–1.19]	**2.44** [1.53–3.78]	**2.07** [1.01–3.90]	0.85 [0.54–1.30]	1.36 [0.77–2.27]
3	119 (5.2%)	**5.74** [3.13–10.13]	**2.56** [1.59–4.01]	**1.80** [1.22–2.63]	1.38 [0.75–2.36]	**2.90** [1.48–5.30]	1.51 [0.99–2.27]	**2.02** [1.19–3.28]
≥4	240 (10.5%)	**10.20** [86.73–15.55]	**3.19** [2.27–4.44]	**1.68** [1.26–2.23]	1.52 [1.00–2.25]	**3.75** [2.37–5.83]	**1.72** [1.28–2.31]	**3.99** [2.87–5.52]
**ACEs (*****N*** **=** **2,288)**								
Emotional abuse	363 (15.9%)	**5.21** [3.74–7.25]	**2.45** [1.85–3.23]	**1.72** [1.35–2.17]	1.39 [0.99–1.92]	**2.87** [1.95–4.16]	**1.66** [1.30–1.12]	**2.81** [2.11–3.71]
Physical abuse	288 (12.6%)	**4.53** [3.19–6.39]	**3.07** [2.28–4.10]	**2.13** [1.65–2.75]	0.97 [0.64–1.42]	**3.48** [2.34–5.10]	**2.12** [1.62–2.75]	**2.56** [1.87–3.46]
Sexual abuse	86 (3.8%)	**6.35** [3.82–10.28]	**2.61** [1.55–4.25]	**1.88** [1.20–2.92]	1.60 [0.85–2.81]	1.95 [0.89–3.80]	0.80 [0.45–1.35]	**2.83** [1.70–4.57]
Emotional neglect	283 (12.4%)	**5.27** [3.73–7.42]	**2.40** [1.76–3.24]	1.28 [0.98–1.67]	1.07 [0.71–1.56]	**2.44** [1.59–3.67]	**1.47** [1.11–1.93]	**2.15** [1.56–2.94]
Physical neglect	107 (4.7%)	**4.94** [3.04–7.82]	**2.83** [1.67–4.05]	**2.19** [1.47–3.25]	1.13 [0.59–1.98]	**2.34** [1.22–4.16]	**1.80** [1.18–2.69]	**3.35** [2.15–5.12]
Parental separation/divorce	324 (14.2%)	**2.80** [1.94–3.97]	**1.77** [1.30–2.39]	0.84 [0.64–1.10]	**1.75** [1.25–2.41]	**1.98** [1.28–2.96]	0.99 [0.75–1.31]	**2.03** [1.49–2.75]
Witnessing domestic abuse	150 (6.6%)	**4.03** [2.59–6.13]	**2.72** [1.84–3.97]	**1.92** [1.36–2.69]	1.18 [0.70–1.91]	**3.49** [2.12–5.55]	**1.71** [1.19–2.43]	**2.58** [1.72–3.78]
Case of addiction in household	285 (12.5%)	**3.68** [2.56–5.23]	**2.14** [1.56–2.91]	**1.46** [1.12–1.89]	**1.84** [1.30–2.57]	**3.27** [2.19–4.82]	1.15 [0.86–1.52]	**3.10** [2.29–4.17]
Mental illness in household	179 (7.8%)	**3.39** [2.21–5.10]	**1.70** [1.13–2.49]	0.84 [0.59–1.18]	1.35 [0.84–2.07]	1.64 [0.92–2.76]	0.83 [0.56–1.20]	**2.07** [1.40–3.00]
Incarceration of familymember	67 (2.9%)	**6.03** [3.40–10.32]	**3.45** [2.02–5.75]	**2.00** [1.21–3.28]	**2.01** [1.03–3.63]	**3.73** [1.85–6.92]	1.39 [0.80–2.34]	**4.45** [2.62–7.40]

*Significant (p ≤ 0.05) odds ratios in bold*.

a *Depression according to PHQ-2 cut-off at ≥ 3*.

b *Lower level of education includes graduates from secondary general school (Volks- and Hauptschule)*.

c *Higher risk of unemployment contains those respondents who were unemployed for at least three times*.

Looking at the economic burdens, we found a higher risk of unemployment within the high-risk group of 4+ ACEs (OR = 3.99). Having reported four or more ACEs was also highly associated with having an equivalized income of under 1,126€ and current unemployment (OR = 3.19 and OR = 3.75). For individual ACEs, the ratios ranged between OR = 1.70 (mental illness in household) and OR = 3.45 (incarceration of family member) for the equivalized income of under 1,126€ and between OR = 1.98 (parental separation or divorce) and OR = 3.73 (incarceration of family member) for current unemployment. Working a part-time job was associated with the incarceration of a family member (OR = 2.01), parental separation or divorce (OR = 1.75) and drug or alcohol abuse in household (OR = 1.84).

### Cumulative effect of ACEs on depression and economic burdens

When put in linear structural equation models, we find significant associations between depression and all economic burdens, except for low level of education and part-time working (see Tables 3, 4). The coefficients ranged from *B* = 0.017 (*p* = 0.031, [0.002–0.032]) for working a blue-collar job to *B* = 0.034 (*p* < 0.000, [0.021–0.047]) for having a low income. Furthermore, both reporting 3 and 4+ ACEs was significantly associated with reporting any economic burden, with an increased effect of 4+ ACEs compared to 3 ACEs. The largest coefficients amongst participants who reported three ACEs were found for low income (*B* = 0.092, *p* = 0.005, [0.028–0.156]) and low level of education (*B* = 0.119, *p* = 0.006, [0.034–0.204]). Working a blue collar or part-time job and experiencing a higher risk of unemployment or being currently unemployed were not significantly associated with reporting three ACEs. The largest coefficients amongst participants who reported four or more ACEs were found for low level of education (*B* = 0.097, *p* = 0.004, [0.031–0.163]), low income (*B* = 0.108, *p* < 0.001, [0.058–0.158]), and higher risk of unemployment (*B* = 0.155, *p* < 0.001, [0.107–0.203]). Only part-time working was not significantly associated with reporting four or more ACEs, but was associated with reporting 2 ACEs (*B* = 0.111, *p* < 0.001, [0.056–0.165]).

**Table 3:** Results of the structural equation models for low income, low level of education and part-time working with 95% confidence intervals.

	**Low income (*****N*** = **2,232)**				**Low level of education**^**a**^ **(*****N*** = **2,261)**				**Part-time working (*****N*** = **2,267)**			
	**Model 1** ***CFI = 1.000*** *** TLI = 1.000*** *** RMSEA = 0.000*** *** SRMR =0.000***		**Model 2** ***CFI = 0.992*** *** TLI = 0.945*** *** RMSEA = 0.029*** *** SRMR = 0.008***		**Model 1** ***CFI = 1.000*** *** TLI = 1.000*** *** RMSEA = 0.000*** *** SRMR = 0.000***		**Model 2** ***CFI = 0.990*** *** TLI = 0.933*** *** RMSEA = 0.028*** *** SRMR = 0.008***		**Model 1** ***CFI = 1.000*** *** TLI = 1.000*** *** RMSEA = 0.000*** *** SRMR = 0.000***		**Model 2** ***CFI = 0.994*** *** TLI = 0.960*** *** RMSEA = 0.024*** *** SRMR = 0.007***	
	***Coef*.**	***p*-Value**	***Coef*.**	***p*-Value**	***Coef*.**	***p*-Value**	***Coef*.**	***p*-Value**	***Coef*.**	***p*-Value**	***Coef*.**	***p*-Value**
**Number of ACEs**												
1 ACE	0.176 (0.055) [−0.027 to 0.380]	0.090	0.033 (0.031) [−0.012 to 0.078]	0.145	−0.024 (−0.017) [−0.084 to 0.036]	0.429	−0.025 (−0.018) [−0.085 to 0.035]	0.410	0.021 (0.021) [−0.021 to 0.062]	0.331	0.024 (0.024) [−0.017 to 0.065]	0.245
2 ACEs	0.167 (0.038) [−0.104 to 0.437]	0.227	0.027 (0.018) [−0.034 to 0.088]	0.384	−0.056 (−0.029) [−0.136 to 0.025]	0.173	−0.059 (−0.031) [−0.139 to 0.022]	0.153	0.106 (0.080) [0.050 to 0.161]	**0.000**	**0.111 (0.084)** [0.056 to 0.165]	**0.000**
3 ACEs	**0.408 (0.089)** [0.138 to 0.677]	**0.003**	**0.092 (0.059)** [0.028 to 0.156]	**0.005**	**0.123 (0.061)** [0.038 to 0.208]	**0.005**	**0.119 (0.059)** [0.034 to 0.204]	**0.006**	0.025 (0.018) [−0.034 to 0.084]	0.405	0.034 (0.024) [−0.024 o 0.092]	0.246
4+ ACEs	**0.476 (0.141)** [0.272 to 0.679]	**0.000**	**0.108 (0.096)** [0.058 to 0.158]	**0.000**	**0.102 (0.069)** [0.037 to 0.168]	**0.002**	**0.097 (0.066)** [0.031 to 0.163]	**0.004**	0.034 (0.033) [−0.011 to 0.079]	0.143	0.028 (0.028) [−0.016 to 0.073]	0.213
Depression	**0.135 (0.154)** [0.084 to 0.185]	**0.000**	**0.034 (0.116)** [0.021 to 0.047]	**0.000**	0.009 (0.023) [−0.008 to 0.026]	0.309	0.009 (0.024) [−0.008 to 0.026]	0.284	0.006 (0.024) [−0.005 to 0.018]	0.293	0.004 (0.014) [−0.008 to 0.015]	0.539
**Moderation**												
Depression × 1 ACE	**0.065 (0.020)** [0.035 to 0.095]	**0.000**	**0.017 (0.015)** [0.009 to 0.024]	**0.004**	0.004 (0.003) [−0.004 to 0.013]	0.314	0.005 [−0.004 to 0.013]	0.290	0.003 (0.003) [−0.003 to 0.009]	0.299	0.002 (0.002) [−0.004 to 0.008]	0.540
Depression × 2 ACEs	**0.094 (0.022)** [0.053 to 0.135]	**0.000**	**0.024 (0.016)** [0.013 to 0.035]	**0.006**	0.006 (0.003) [−0.006 to 0.018]	0.314	0.006 [−0.005 to 0.018]	0.289	0.004 (0.003) [−0.004 to 0.013]	0.299	0.003 (0.002) [−0.006 to 0.011]	0.540
Depression × 3 ACEs	**0.110 (0.024)** [0.063 to 0.157]	**0.000**	**0.028 (0.018)** [0.015 to 0.041]	**0.006**	0.007 (0.003) [−0.007 to 0.021]	0.313	0.007 [−0.006 to 0.021]	0.288	0.005 (0.004) [−0.004 to 0.015]	0.298	0.003 (0.002) [−0.006 to 0.012]	0.540
Depression × 4+ ACEs	**0.174 (0.052)** [0.107 to 0.240]	**0.000**	**0.044 (0.039)** [0.027 to 0.061]	**0.009**	0.011 (0.008) [−0.010 to 0.033]	0.310	0.012 [−0.010 to 0.033]	0.285	0.008 (0.008) [−0.007 to 0.023]	0.294	0.005 (0.004) [−0.010 to 0.019]	0.539
**Total effect of ACEs moderated by depression**												
Depression × 1 ACE	**0.241 (0.075)** [0.037 to 0.446]	**0.021**	**0.050 (0.046)** [0.005 to 0.095]	**0.023**	−0.020 (−0.014) [−0.079 to 0.039]	0.512	−0.021 [−0.080 to 0.039]	0.496	0.024 (0.024) [−0.017 to 0.065]	0.257	0.026 (0.026) [−0.014 to 0.066]	0.207
Depression × 2 ACEs	0.261 (0.060) [−0.011 to 0.532]	0.060	**0.051 (0.035)** [−0.010 to 0.111]	**0.031**	−0.050 (−0.026) [−0.129 to 0.030]	0.220	−0.052 [−0.132 to 0.027]	0.198	**0.110 (0.083)** [0.055 to 0.165]	**0.000**	**0.113 (0.085)** [0.059 to 0.167]	**0.000**
Depression × 3 ACEs	**0.518 (0.113)** [0.252 to 0.783]	**0.000**	**0.120 (0.077)** [0.056 to 0.183]	**0.032**	**0.130 (0.064)** [0.046 to 0.214]	**0.002**	**0.126** [0.042 to 0.210]	**0.003**	0.030 (0.021) [−0.028 to 0.089]	0.311	0.037 (0.026) [−0.020 to 0.0.94]	0.202
Depression × 4+ ACEs	**0.649 (0.193)** [0.456 to 0.842]	**0.000**	**0.152 (0.134)** [0.105 to 0.199]	**0.024**	**0.114 (0.077)** [0.052 to 0.175]	**0.000**	**0.109** [0.047 to 0.171]	**0.001**	0.042 (0.041) [−0.001 to 0.084]	0.055	0.033 (0.032) [−0.009 to 0.075]	0.126
**Control variables**												
Gender (Ref.: Men)			0.026 (0.038) [−0.002 to 0.054]	0.068			−0.015 (−0.016) [−0.052 to 0.022]	0.433			**0.133 (0.211)** [0.108–0.158]	**0.000**
Migration background			**0.114 (0.106)** [0.070 to 0.157]	**0.000**			0.047 (0.033) [−0.011 to 0.105]	0.114			−0.003 (−0.004) [−0.043 to 0.036]	0.864

*Significant (p ≤ 0.05) coefficients in bold*.

Standardized coefficient and 95% confidence interval in brackets.

a *Lower level of education includes graduated from secondary general school (Volks- and Hauptschule)*.

**Table 4:** Results of the structural equation models for current unemployment, blue collar employment and higher risk of unemployment with 95% confidence intervals.

	**Currently unemployed (*****N*** = **2,267)**				**Blue collar job (*****N*** = **2,255)**				**Higher risk of unemployment**^**a**^ **(*****N*** = **2,249)**			
	**Model 1** ***CFI = 1.000*** *** TLI = 1.000*** *** RMSEA = 0.000*** ***SRMR = 0.000***		**Model 2** ***CFI = 0.994*** *** TLI = 0.959*** *** RMSEA = 0.024*** ***SRMR = 0.007***		**Model 1** ***CFI = 1.000*** *** TLI = 1.000*** *** RMSEA = 0.000*** ***SRMR = 0.000***		**Model 2** ***CFI = 0.994*** *** TLI = 0.959*** *** RMSEA = 0.029*** ***SRMR = 0.008***		**Model 1** ***CFI = 1.000*** *** TLI = 1.000*** *** RMSEA = 0.000*** ***SRMR = 0.000***		**Model 2** ***CFI = 0.991*** *** TLI = 0.942*** *** RMSEA = 0.029*** ***SRMR = 0.008***	
	***Coef*.**	***p*-Value**	***Coef*.**	***p*-Value**	***Coef*.**	***p*-Value**	***Coef*.**	***p*-Value**	***Coef*.**	***p*-Value**	***Coef*.**	***p*-Value**
**Number of ACEs**												
1 ACE	0.006 (0.008) [−0.025 to 0.037]	0.712	0.005 (0.006) [−0.026 to 0.035]	0.771	−0.011 (−0.008) [−0.067 to 0.046]	0.715	−0.019 (−0.014) [−0.072 to 0.035]	0.495	0.016 (0.015) [−0.028 to 0.060]	0.478	0.015 (0.014) [−0.028 to 0.059]	0.494
2 ACEs	0.019 (0.019) [−0.022 to 0.060]	0.367	0.016 (0.016) [−0.025 to 0.057]	0.438	−0.032 (−0.018) [−0.108 to 0.044]	0.410	−0.043 (−0.024) [−0.115 to 0.029]	0.239	0.008 (0.006) [−0.051 to 0.066]	0.793	0.004 (0.003) [−0.054 to 0.062]	0.897
3 ACEs	**0.044 (0.042)** [0.000 to 0.088]	**0.049**	0.040 (0.037) [−0.004 to 0.083]	0.078	0.073 (0.038) [−0.008 to 0.154]	0.078	0.050 (0.026) [−0.027 to 0.126]	0.204	0.056 (0.037) [−0.006 to 0.118]	0.079	0.050 (0.033) [−0.012 to 0.112]	0.115
4+ ACEs	**0.058 (0.076)** [0.025 to 0.091]	**0.001**	**0.055 (0.072)** [0.022 to 0.089]	**0.001**	**0.097 (0.069)** [0.035 to 0.159]	**0.002**	**0.103 (0.074)** [0.044 to 0.162]	**0.001**	**0.165 (0.151)** [0.117 to 0.212]	**0.000**	**0.155 (0.142)** [0.107 to 0.203]	**0.000**
Depression	**0.031 (0.155)** [0.023 to 0.040]	**0.000**	**0.032 (0.159)** [0.023 to 0.041]	**0.000**	0.010 (0.029) [−0.006 to 0.026]	0.203	**0.017 (0.046)** [0.002 to 0.032]	**0.031**	**0.030 (0.107)** [0.018 to 0.043]	**0.000**	**0.031 (0.109)** [0.019 to 0.043]	**0.000**
**Moderation**												
Depression × 1 ACE	**0.016 (0.021)** [0.009 to 0.022]	**0.000**	**0.016 (0.022)** [0.010 to 0.022]	**0.000**	0.005 (0.004) [−0.003 to 0.013]	0.211	0.008 (0.006) [0.000 to 0.016]	**0.040**	**0.015 (0.015)** [0.008 to 0.023]	**0.000**	**0.016 (0.015)** [0.008 to 0.023]	**0.000**
Depression × 2 ACEs	**0.022 (0.022)** [0.013 to 0.030]	**0.000**	**0.022 (0.022)** [0.014 to 0.031]	**0.000**	0.007 (0.004) [−0.004 to 0.018]	0.211	**0.011 (0.006)** [0.001 to 0.022]	**0.040**	**0.021 (0.015)** [0.011 to 0.032]	**0.000**	**0.022 (0.015)** [0.011 to 0.032]	**0.000**
Depression × 3 ACEs	**0.025 (0.024)** [0.016 to 0.034]	**0.000**	**0.026 (0.024)** [0.016 to 0.035]	**0.000**	0.008 (0.004) [−0.005 to 0.022]	0.209	**0.014 (0.007)** [0.001 to 0.026]	**0.038**	**0.025 (0.016)** [0.013 to 0.036]	**0.000**	**0.025 (0.017)** [0.013 to 0.037]	**0.000**
Depression × 4+ ACEs	**0.039 (0.051)** [0.027 to 0.051]	**0.000**	**0.040 (0.052)** [0.028 to 0.052]	**0.000**	0.013 (0.009) [−0.007 to 0.034]	0.205	**0.021 (0.015)** [0.002 to 0.040]	**0.032**	**0.039 (0.035)** [0.022 to 0.055]	**0.000**	**0.039 (0.036)** [0.023 to 0.055]	**0.000**
**Total Effect of ACEs moderated by depression**												
Depression × 1 ACE	0.021 (0.029) [−0.009 to 0.052]	0.172	0.021 (0.028) [−0.010 to 0.051]	0.189	−0.005 (−0.004) [−0.062 to 0.051]	0.850	−0.010 (−0.008) [−0.064 to 0.043]	0.701	0.031 (0.030) [−0.012 to 0.075]	0.160	0.031 (0.029) [−0.013 to 0.074]	0.165
Depression × 2 ACEs	0.041 (0.041) [−0.000 to 0.082]	0.052	0.038 (0.039) [−0.002 to 0.079]	0.066	−0.025 (−0.014) [−0.100 to 0.050]	0.517	−0.032 (−0.018) [−0.103 to 0.039]	0.382	0.029 (0.021) [−0.029 to 0.087]	0.325	0.025 (0.018) [−0.032 to 0.083]	0.389
Depression × 3 ACEs	**0.069 (0.065)** [0.025 to 0.113]	**0.002**	**0.065 (0.062)** [0.022 to 0.109]	**0.003**	**0.081 (0.042)** [0.001 to 0.161]	**0.046**	0.063 (0.033) [−0.013 to 0.139]	0.102	**0.080 (0.053)** [0.019 to 0.142]	**0.011**	**0.075 (0.050)** [0.013 to 0.136]	**0.017**
Depression × 4+ ACEs	**0.097 (0.127)** [0.065 to 0.129]	**0.000**	**0.095 (0.124)** [0.063 to 0.127]	**0.000**	**0.110 (0.079)** [0.051 to 0.168]	**0.000**	**0.124 (0.089)** [0.068 to 0.180]	**0.000**	**0.203 (0.186)** [0.158 to 0.248]	**0.000**	**0.194 (0.178)** [0.149 to 0.239]	**0.000**
**Control variables**												
Gender (Ref.: Men)			–**0.032** (–**0.068)** [−0.051 to −0.013]	**0.001**			–**0.278** (–**0.325)** [−0.312 to −0.245]	**0.000**			−0.007 (−0.010) [−0.034 to 0.020]	0.610
Migration background			**0.035 (0.047)** [0.005 to 0.065]	**0.022**			0.025 (0.019) [−0.027 to 0.077]	0.352			**0.081 (0.077)** [0.038 to 0.123]	**0.000**

*Significant (p ≤ 0.05) coefficients in bold*.

*Standardized coefficient and 95% confidence interval in brackets*.

a *Higher risk of unemployment contains those respondents who were unemployed for at least three times*.

The interaction terms between depression and number of ACEs were mostly significant, except for the cases of low level of education and part-time working. We found the largest moderation effects amongst those who reported four or more ACEs, indicating that demonstrating signs of depression amongst this population increased the total effect of number of ACEs by up to *B* = 0.044 (low income, *p* = 0.009, [0.027–0.061]). Additionally, we also found gender effects as well as effects of having a migration background for almost all economic burdens when including gender and migration as control variables. Being a woman decreased the risk of being unemployed (*B* = −0.032, *p* = 0.001, (−0.051 to −0.013)] and working in a blue-collar job (*B* = −0.278, *p* < 0.001, [−0.312 to −0.245]), but increased the risk of working part-time (*B* = 0.133, *p* < 0.001, [0.108–0.158]). Having a migration background increased the risk of being currently unemployed (*B* = 0.035, *p* = 0.022, [0.005–0.065]) and being at greater risk of unemployment in general (*B* = 0.081, *p* < 0.001, [0.038–0.123]), as well as having a lower income (*B* = 0.114, *p* < 0.001, [0.070–0.157]).

## Discussion

In this study, we investigated the (co-)occurrence of ACEs and their correlations to negative economic outcomes, such as lower income, lower level of education, type of occupation and unemployment, as well as depression. In total, 33.2% reported at least one ACE, including 10.5% reporting four or more. The most common ACE was emotional abuse, followed by parental separation or divorce. Least common were incarceration of a family member and sexual abuse. These findings were largely in line with other findings of representative studies in Germany, in which the prevalence of child maltreatment ranged from 31.0% (22) to 43.7% (1). The prevalence of adverse childhood experiences mirrored findings from a previous representative cross-sectional study of German samples (1, 2, 37), in which the frequencies for physical and emotional abuse were reported to be significantly higher and frequencies for sexual abuse were reported significantly lower than those of previous studies (22, 37, 38).

### Association with depression and role as moderator

We found that each individual ACE showed a strong association to depression with odds ratios ranging from OR = 2.80 (parental separation) to OR = 6.35 (sexual abuse). These findings were significantly higher than those of, for example, Witt et al. (1), who found (unadjusted) odds ranging from OR = 2.04 (parental separation) to OR = 3.94 (sexual abuse). The relatively high odds for depressive symptoms after experiencing sexual trauma in childhood is a common and widely discussed phenomenon in literature (39, 40). Though, more recent studies conclude, above all, neglect and emotional abuse to have the strongest and the most relevant associations to depression while sexual and physical abuse may be less strongly associated (40). The association between adverse experiences and depression might be result of a higher sensitivity to stress, a heightened emotional vulnerability and engagement with repetitive negative thinking after the experience of adverse events in childhood (13, 14, 16).

We found that having experienced 3 or 4+ ACEs increased the risk for depressive symptoms. This result is in accordance with previous studies indicating an association between cumulative ACEs and negative psychological outcomes (1, 23, 39). Not only were the odds of depression higher amongst those who reported multiple, or more specifically 4+ ACEs, even after controlling for gender and migration background. This might be due to negative metacognitive beliefs resulting from adverse experiences in childhood that are associated with high levels of depression. Researchers have found that a low confidence in one's own memory was associated with low work ability (41). When adjusting in a structural equation model, the significant associations for depression were lowest for working a blue-collar job and highest for being currently unemployed. Depression had no significant association with low level of education and part-time working, indicating that depression, on itself, does not increase the risk of those factors (3).

As for economic burdens and the occurrence of ACEs, we found a generally higher risk of occurrence of economic burdens in one's life the more ACEs the person has experienced, except for people who worked part-time. Respondents who suffered four or more ACEs, for example, were more likely to be at risk of poverty with an equivalized income of under 1,126€ and a blue-collar job. Furthermore, they were also more likely to be currently unemployed and to be at greater risk of unemployment, in general. This mirrors results from previous studies (4, 6, 18–22, 42). Additionally, when investigating the moderating effect of depression, we found that almost all interaction terms were significantly moderating and increasing the effects of number of ACEs on economic burdens. The largest effects were found amongst those who reported four or more ACEs, further cementing the negative cumulative impact of ACEs on adult economic life. Our findings on the relation of ACEs, depression and economic performance in adult life have serious clinical implications: The importance of the role of depression after having experienced childhood adversities on adulthood economic outcomes, it is crucial to be able to identify possible depressive symptoms early in order to induce psychological intervention, such as, for example, metacognitive psychotherapy (14, 43).

### The role of gender and migration background

Finally, the role of gender needs to be addressed. We found a lower likelihood for women to be affected by current unemployment or working a blue-collar job compared to men. Instead, we found that women were more likely to live at risk of poverty with an equivalized income of under 1,126€ and to work part-time jobs. This might be due to gender roles in traditional family constellations where women typically work part-time jobs in addition to caring for children while their husband is typically working full-time. This might also explain the lower income and the insignificant results for part-time working in the structural equation models for ACEs.

Additionally, we have to consider migration background as an important factor when investigating economic burdens. Our analysis showed a significant association between having a migration background and an increased risk of being unemployed or being at risk of poverty. As this analysis showed by the increasing moderation effect of depression, this association might be due to the increased risks of depressive symptoms in those with migration background compared to non-migrants (44–46). However, this discrepancy in depression prevalence has been heavily disputed by contradicting findings from other researchers who found no differences between migrants and German natives [see for example (47, 48)]. Self-attribution of being a migrant might also play a role in demonstrating depressive symptoms (48). It also needs to be mentioned that these economic burdens can also be affect by one's own socioeconomic status as a child: A lower socioeconomic status is not only associated with the prevalence of ACEs, it also affects the likelihood of a person having a lower education and, in turn, work a lower paid job (23, 49, 50).

### Strengths and limitations

A strength of this study is the large and representative sample size of 2,288 participants. To measure adverse childhood experiences, we used the well-established ACE questionnaire. This measure has been discussed because of its reliability (since it's a retrospective self-reporting measure) and bias (23). However, the biggest benefit of using the ACE questionnaire is that it leaves little room for misinterpretation with its binary questions. The possibility of false-negative statements due to suppression or concealment out of shame has also been discussed extensively in the literature [see for example Harft (51)]. Furthermore, it is possible that this sample excludes participants who are at a higher risk of experiencing sexual trauma, as previous studies showed higher rates of sexual abuse (52). And finally, as previous research showed, childhood maltreatment can be “both a cause and consequence of poverty” [(23), p. 122], making it difficult to test for a definite causal relationship, especially with cross-sectional data. In the same vein, a high risk of being unemployed and generally being more vulnerable to unemployment when suffering depression has been widely discussed (53, 54). Though, it might also be plausible that unemployed respondents suffer from depressive symptoms because of their unemployment, as evidence from many studies leads to suggest (55, 56).

## Data availability statement

The data analyzed in this study is subject to the following licenses/restrictions. The dataset is not publicly available. Requests to access these datasets should be directed at: EB, Elmar.Braehler@medizin.uni-leipzig.de.

## Ethics statement

The studies involving human participants were reviewed and approved by the Ethics Committee of the Medical Department of the University of Leipzig (Ref. No. 474/20-ek). Written informed consent to participate in this study was provided by the participants' legal guardian/next of kin.

## Author contributions

EB, JF, CS, and MB were involved in the study design. JP performed the statistical analysis and wrote the manuscript. A-CS, EB, MB, JF, and CS provided ideas as well as guidance and critical feedback to the final version of the manuscript. All authors contributed to the article and approved the submitted version.

## Conflict of interest

The authors declare that the research was conducted in the absence of any commercial or financial relationships that could be construed as a potential conflict of interest.

## Publisher's note

All claims expressed in this article are solely those of the authors and do not necessarily represent those of their affiliated organizations, or those of the publisher, the editors and the reviewers. Any product that may be evaluated in this article, or claim that may be made by its manufacturer, is not guaranteed or endorsed by the publisher.
